# Case Report: Surgical thrombectomy in a patient with isolated cortical vein thrombosis previously misdiagnosed as brain tumor

**DOI:** 10.3389/fonc.2022.977038

**Published:** 2022-11-01

**Authors:** Yang Bai, Liansheng Dong, Chunyong Yu, Sizhe Feng, Guobiao Liang

**Affiliations:** ^1^ Department of Neurosurgery, General Hospital of Northern Theater Command, Shenyang, China; ^2^ Department of Neurosurgery, Ansteel General Hospital, Anshan, China

**Keywords:** isolated cortical vein thrombosis, thrombectomy, brain tumor, neuroimaging, diagnosis and treatment

## Abstract

**Introduction:**

As a rare type of cerebral venous thrombosis, isolated cortical vein thrombosis (ICVT) is easily misdiagnosed as brain tumor, especially in the cases with prominent signs of parenchymal brain lesions. Despite controversy concerning the efficacy and safety, anticoagulant treatment dominates in current therapeutic strategies for ICVT. As yet, surgical thrombectomy in the treatment of ICVT has not been reported. We present hereafter a female with ICVT previously misdiagnosed as brain tumor who had successful surgical thrombectomy.

**Case description:**

A 54-year-old female with progressive left-sided limb weakness suddenly developed focal tonic-clonic epileptic seizure. Physical examination indicated strength of 0/5 in the left limbs. Magnetic resonance imaging (MRI) showed an irregular juxtacortical lesion surrounded with massive edema in the frontoparietal cortex, which was initially diagnosed as glioma. However, it turned out to be ICVT of the central sulcus vein during craniotomy. Then, venotomy and thrombectomy were performed, with instant recanalization of the vein noticed during surgery. In retrospect, we identified the suspected ICVT of the central sulcus vein in preoperative magnetic resonance venotography (MRV) and contrast MRI images. Laboratory tests also revealed homocysteinemia and hypercoagulable states in the patient. Follow-up MRV obtained 3 months after discharge showed cortical vein recanalization. At the one-year follow-up, she exhibited subtle sequelae of weakness in the left lower limb with a modified Rankin scale score of 1.

**Discussion:**

Physicians should be aware of ICVT in the differential diagnoses in patients with risk factors, classical symptoms, and parenchymal brain lesions in or near cortex. Surgical thrombectomy excels at realizing definite recanalization and avoiding systematic complications of anticoagulation. It might be a therapeutic alternative for ICVT, especially when craniotomy is performed for treating intracranial hypertension or a definite diagnosis is made during craniotomy.

## Introduction

As a rare form of cerebral venous thrombosis (CVT) without sinus or deep venous involvement, isolated cortical vein thrombosis (ICVT) has only been reported in single case or small series (1). Despite overall favorable prognosis, a timely diagnosis is pivotal since this disease has the potential to develop permanent parenchymal damage (2). However, the diagnosis of ICVT is more challenging compared with CVT due to its widely variable clinico-radiological spectrum (3). Typical clinical presentations are headache, seizures, and focal neurologic deficits (4), but syndromes owing to increased intracranial pressure should not be overlooked (5, 6). The gold standard of imaging diagnosis of ICVT is the visualization of the thrombosed vein (i.e., the cord sign). However, this is frequently unapproachable because of the great variability in cortical veins and difficulty of current imaging in identifying small occluded vessels (7). This condition is further complicated by signs of parenchymal lesions secondary to vasogenic edema, intraparenchymal hemorrhage, and venous infarction, reminiscent of those seen in intracranial space-occupying lesions. Thus, among the scant reported cases, ICVT misdiagnosed as brain space-occupying lesion is not seldom encountered. In these cases, the diagnosis of ICVT was only confirmed intraoperatively (5, 6, 8).

The therapy for ICVT follows the treatment guideline for sinus vein thromboses (9). For patients in stable condition, anticoagulation is recommended even in the presence of cerebral hemorrhage. For those with disease deterioration, the management of intracranial hypertension should be addressed, and this is the case of hematoma evacuation and decompressive craniotomy in previous literature (8, 10, 11). However, surgical venotomy and thrombectomy has not been reported as yet. Herein, we present the first case of surgical thrombectomy in a patient with ICVT previously misdiagnosed as brain tumor. We hope this report may provide novel diagnostic and therapeutic clues for the management of ICVT.

## Case description

**Figure 1:**
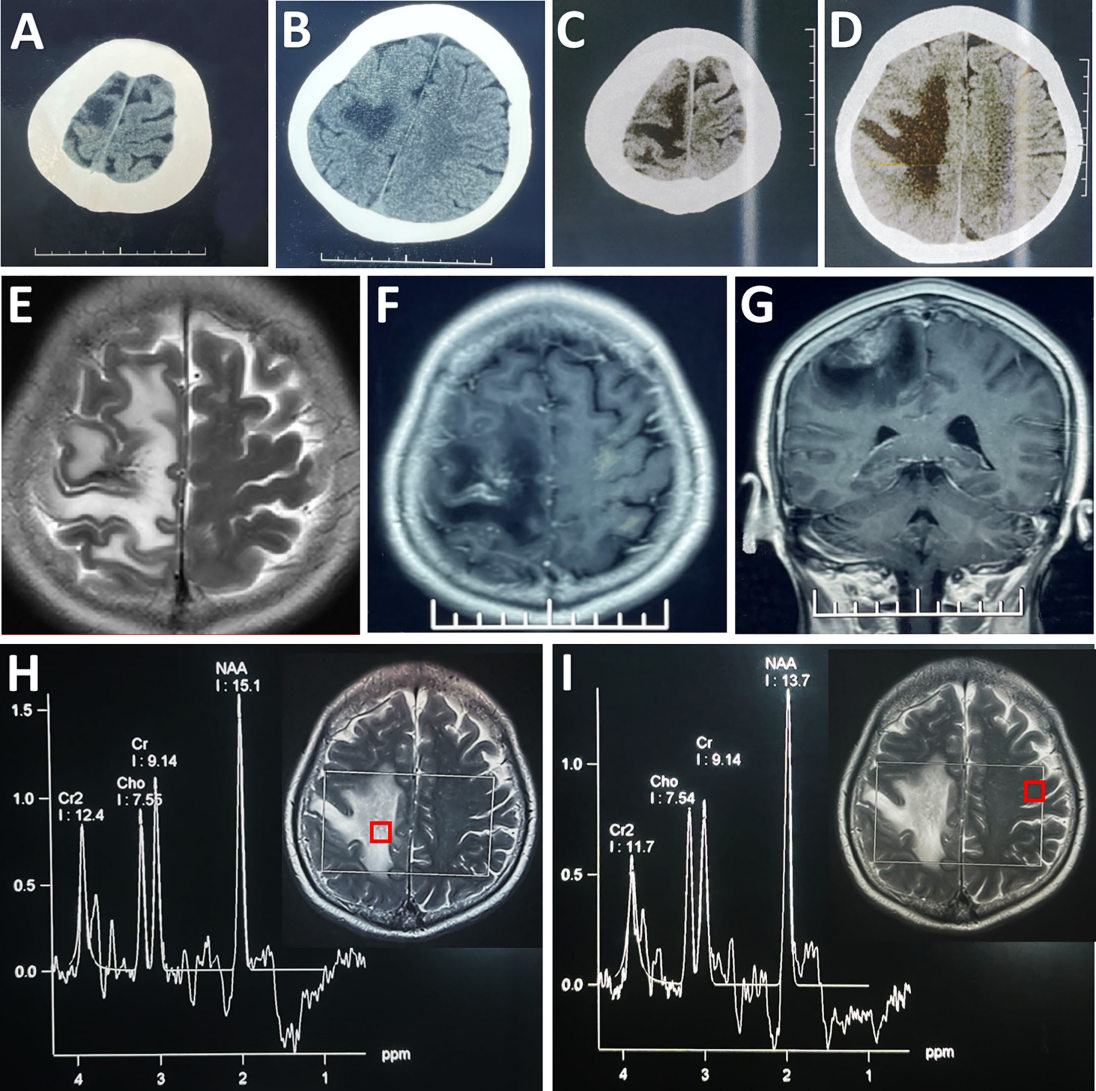
Radiological evaluation of the parenchymal lesion preoperatively. An initial CT scan showed a hypodense irregular mass located in the right frontal cortex **(A, B)**, which enlarged in size in the second scan performed 4 days later **(C, D)**. The lesion was hyper-intense on T2WI **(E)**. Post-contrast (gadolinium-enhanced) axial **(F)** and coronal MRI **(G)** showed prominent heterogeneous enhancement. MRS showed that there was no obvious change in the ratios of NAA/Cr (1.65) and Cho/Cr (0.83) in the lesion **(H)** compared with its counterpart (NAA/Cr: 1.50; Cho/Cr: 0.82) on the contralateral side **(I)**. Each region of interest in the MR images showed the location where the spectrum was obtained.

A 54-year-old postmenopausal women with a one-week history of progressive left-sided limb weakness suddenly developed involuntary tonic-clonic movements involving left-sided limbs. She did not lose consciousness during the seizure. This episode lasted for 40 min before she received antiepileptic drugs and propofol treatment in a local emergency department. An initial computed tomography (CT) revealed a cortical based right frontoparietal hypodense lesion (**Figures 1A, B**), which prompted her presentation to our facility three days later. During the last two years, she had experienced two episodes of tonic-clonic seizures involving the left hand and abdomen without cognitive impairment, each lasting less than 5 minutes. She declared a diagnosis of epilepsy after the first episode in a local hospital, with no obvious signs of brain lesion in CT scans, and recovered with no sequela after conservative therapy. She reported a two-year smoking history, and denied alcohol, drug, and medication use. There was no history of infection, trauma, surgery, and exposure to toxic substances. She had a body mass index of 22.03 kg/m^2^. Upon arrival, she was afebrile with normal vital signs. Neurological examinations revealed power of 0/5 and hyperactive tendon reflexes in the left limbs. Babinski sign in the left-side was positive. Laboratory studies showed that serum liver and kidney function indexes, lipid profile, glucose, ion levels, tumor markers, and complete blood counts were all within normal limits.

**Figure 2:**
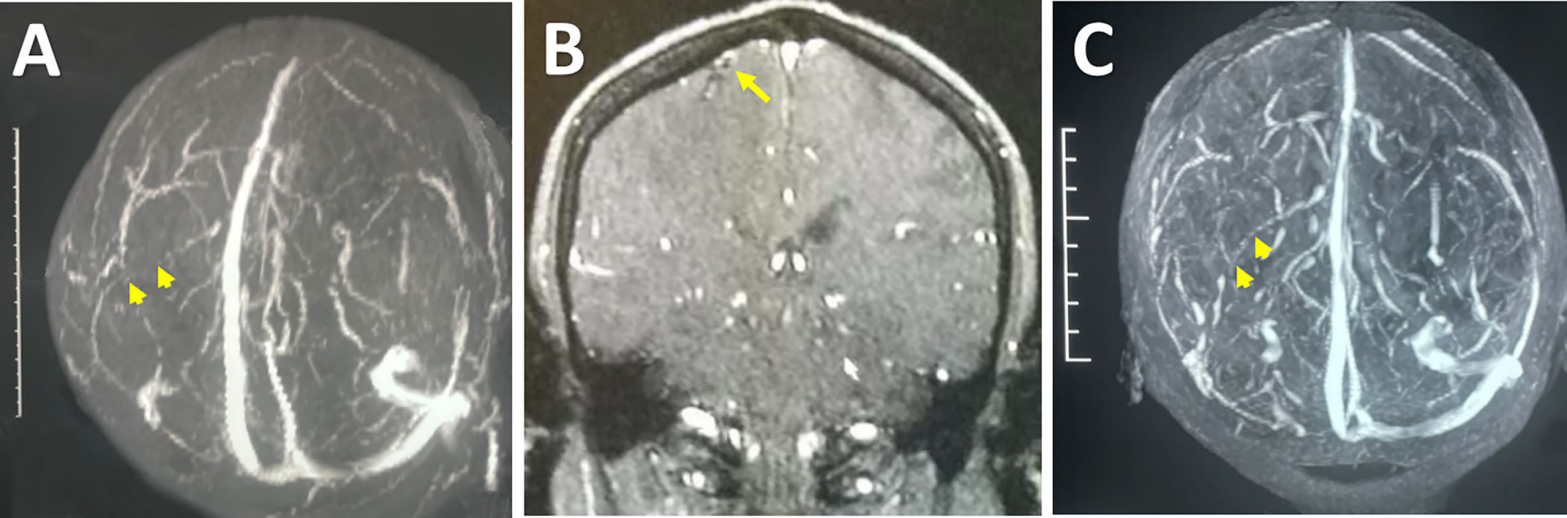
Radiological images indicating the ICVT. Preoperative MRV revealed an absence of flow (arrowheads) in the left central sulcus vein **(A)**. Preoperative contrast MRI showed an intraluminal filling defect at the site where the deep branch of the right central sulcus vein enters the main trunk (arrow), likely indicative of ICVT **(B)**. Follow-up MRV showed the recanalization (arrowheads) of the right central sulcus vein after surgical thrombectomy **(C)**.

A repeated CT scan showed the expansion of the lesions of hypodensity in the right frontoparietal cortex (**Figures 1C, D**). On magnetic resonance (MR) images, the lesion presented high signal intensity on T2-weighted images (**Figure 1E**), and a mixture of hypointensity and isointensity on T1-weighted images. Massive edema surrounding the lesion was noted on T2-weighted and fluid attenuated inversion recovery images. Focal enhancement was observed in the contrast-enhanced MRI (**Figures 1F, G**). Proton MR spectra (MRS) revealed no obvious change in N-acetylaspartate (NAA)/creatine (Cr) ratio or choline/Cr ratio within the lesion compared with contralateral frontal lobe (**Figures 1H, I**). CT angiography (CTA) excluded aneurysm, arteriovenous malformation, and dural arteriovenous fistula. There seemed to be no obvious abnormity in the MR venography (MRV) when assessed pre-operatively (**Figure 2A**).

**Figure 3:**
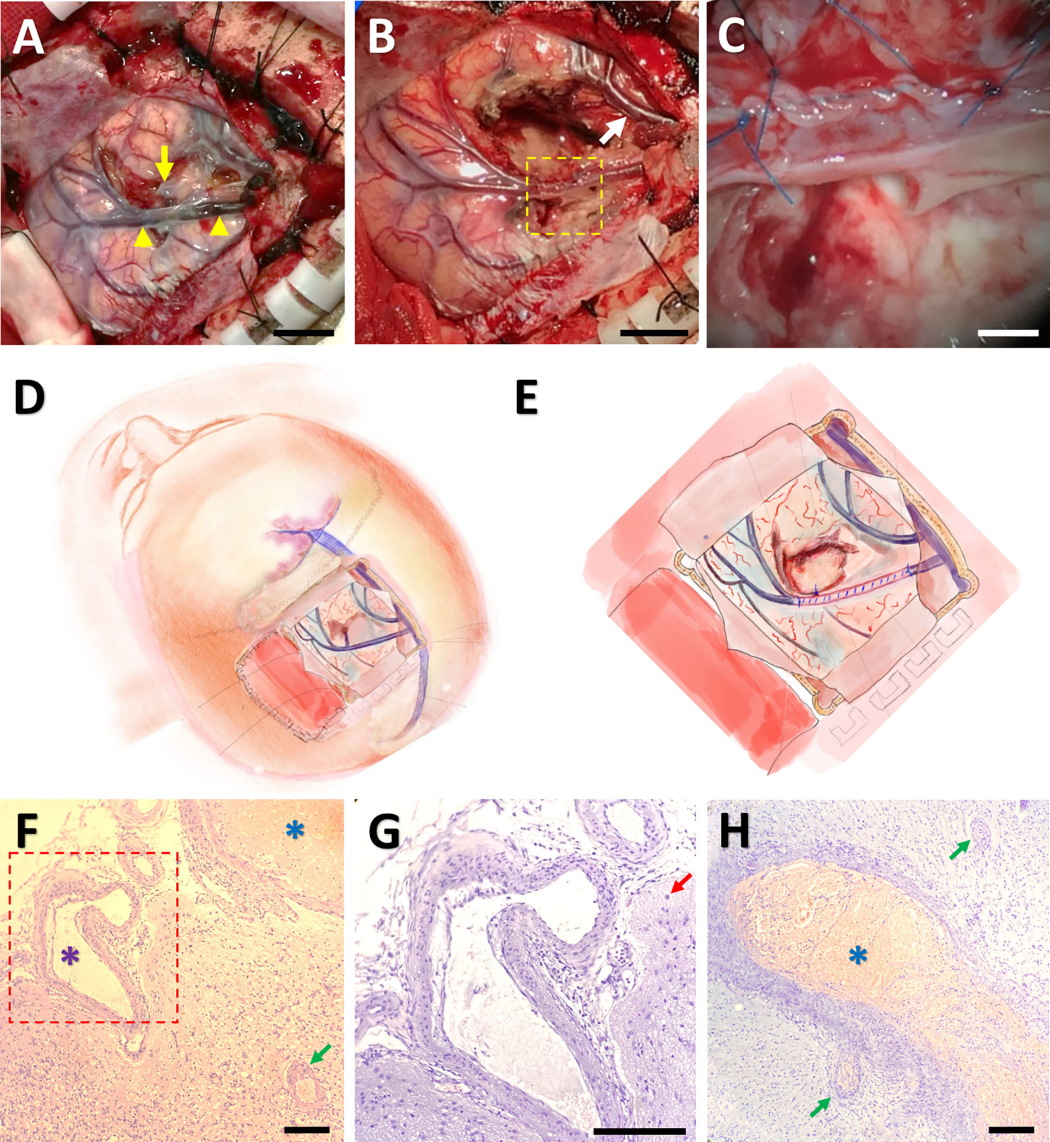
Surgical thrombectomy and pathological confirmation of ICVT. An intraoperative image showing that the cortical vein (indicated by yellow arrowheads) as well as its deep branch (the yellow arrow) became darkened and stiff. The arrowheads denoted the starting and ending points of the thrombosis **(A)**. After surgical thrombectomy, both the occluded vein and an adjacent cortical vein (the white arrow) returned to normal color **(B)**. The framed area in **(B)** was magnified in **(C)**, indicating suture of the vein after thrombus removal. Bars = 1 cm in **(A, B)**, and 200 mm in **(C)** The operation approach **(D)** and surgical thrombectomy **(E)** were further illustrated by cartoon images. Hematoxylin-eosin staining under light microscopy showed thrombosis in some venules (blue arterisks), vascular malformation (the purple arterisk) and proliferation (green arrows), and gliosis (the red arrow) **(F-H)**. The framed area in **(F)** was magnified in **(G)**. Bars = 50 μm in **(F-H)**.

Given these clinic-radiological features, an initial diagnosis was tentatively made as glioma, and craniotomy was performed. Intraoperatively, the central sulcus vein appeared black and stiff, and the surface of the precentral gyrus with the occlusion vein was congested, indicating possible thrombosis of the cortical vein. Under vascular exclusion assisted with cotton-pieces, an incision was made along the longitudinal axis of the vein, and a black 2-centimeter-long thrombus was noticed. This thrombus started approximately 3 centimeters away from the superior sagittal sinus, terminated before the bifurcation of the vein, and spread into the lumen of a deep branch (**Figure 3A**). Then, the thrombus occluding the trunk of the vessel was removed. Next, the lumen of the vein was perfused with heparin in order for preventing thrombosis, and the incision was continuously sutured with 7-0 threads (**Figures 3B, C**). After release of the cotton-pieces, the central sulcus vein returned to normal color, indicating instant successful reflow. Interestingly, an adjacent superficial vein also returned to normal color after thrombectomy, which might be owing to the development of collateral circulation between the two veins after ICVT. Finally, cortical tissue around the occluded deep branch were harvested for pathological examination, and then the branch was sacrificed (**Figures 3B-E**).

Pathological analysis revealed proliferation of small vessels and reactive gliocytes, vascular malformation, and thrombosis in some vessels, with no characteristics of tumour-like lesions observed (**Figures 3F-H**). Retrospectively, we noticed homocysteinemia (45.5 μmol/L; ref: 0-15 μmol/L), as well as hypercoagulable states indicated by high levels of fibrinogen degradation product (25.05 mg/L; ref: 0.01-5.00 mg/L) and D-dimer (5.63 mg/L; ref: 0.01-0.55 mg/L) in this patient. Upon revisiting the imaging data, we identified filling defect in the central sulcus vein (**Figure 2A**) and a suspected site of thrombosis in the deep branch of the central sulcus vein (**Figure 2B**).

Based on the final diagnosis of ICVT, she was treated with intravenous injection of traditional Chinese medicine danshen for improving microcirculation and preventing thrombosis (12, 13). Conventional anticoagulation drugs were not used owing to the risk of postoperative bleeding. After one-month rehabilitation, the patient was discharged with Grade 5 and 4 muscle power in the left upper and lower extremities, respectively. Follow-up MRV obtained 3 months after discharge showed cortical vein recanalization (**Figure 2C**). At her one-year follow-up appointment, she exhibited sequelae of slight weakness in the left lower limb after a long-walk, with a modified Rankin scale score of 1. At a five-year follow-up, the patient denied any symptom recurrence (**Supplemental Figure 1**).

## Discussion

Radiological characteristics of ICVT mainly fall into the cord sign and indirect signs (2, 14). The former is only occasionally seen on CT scans. MRI equivalent of the cord sign is also difficult to identify on conventional T1/T2 images since the signal intensity of venous thrombus is complicated by the interval between the onset of thrombosis and radiologic examinations. It is only discernable in the subacute early phase when the clot signal becomes hyperintense on T1/T2 images. Interestingly, the T2-susceptibility-weighted gradient echo (GE) sequence is sensitive to all paramagnetic products of intraluminal hemoglobin and could provide better delineation of the cord sign. Thus, it becomes the most recognized method for detection of ICVT (15, 16). Digital subtraction angiography (DSA) is recommended as a further diagnostic tool when CT or MRI produces suspicious results. Collateral venous pathways, tortuous veins, delayed local venous drainage, and missing or rarefied contrast of the affected cortical vein are esteemed as positive signs of ICVT (16). With regard to CT venography or MRV, they aid in the identification of sinus involvement but lack adequate sensitivity to accurately detect cortical veins (17). Apart from direct signs, abnormalities in brain parenchyma, including localized edema, hemorrhagic infarction, and intracerebral hemorrhage, are usually present near the affected vein (7). Therefore, the differential diagnosis should be cautiously made with brain space-occupying lesions, especially when direct sign is invisible.

In this case, the direct sign could not be seen on conventional CT and MRI scans, while indirect signs were confined to progressive massive edema. The prominent irregular enhancement on contrast enhanced MRI scans mimicked that seen in the case of brain tumor, which in fact might be extravasation of the contrast agent from the injured vascular epithelium under the condition of long-term venous hypertension. All these characteristics led to a misdiagnosis of advanced malignant tumor. The sole imaging evidence in support of the diagnosis of ICVT was rarefied contrast of the affected cortical vein revealed by pre-operative MRV, but this was only discernable when comparing with the recanalized vessel in the follow-up MRV. It was worth mentioning that the MRS data revealing no change in NAA or choline level did not support the diagnosis of malignant brain tumor. In a previous report of ICVT, MRS revealed decreased NAA levels together with the presence of lactate within the lesion, indicating acute ischemic venous infarction (18). Herein, we presented a case of ICVT without abnormal MRS features, further demonstrating the multi-faceted pathophysiology and imaging characteristics of ICVT.

Apart from anatomical etiologies leading to slow blood flow or reflux (5), acknowledged systemic factors for CVT in all its subtypes include pregnancy, dehydration, drugs, systematic diseases (e.g., coagulopathy, malignancy, autoimmune diseases), and intracranial hypotension (19, 20). In this case, the patient had prothrombotic states that we believe may contribute to the development of ICVT. The molecular basis of prothrombotic states depends on hereditary and acquired factors, among which homocysteine is a key thrombophilic factor (21). Homocysteinemia may provoke thrombotic events *via* platelet aggregation, increased activation of factor V, prothrombin activation, and inhibition of protein C activation (22, 23). Apart from homocysteinemia, hereditary factors, such as prothrombin gene mutation (24), type II protein S deficiency (11), and activated protein C resistance (25), were also involved in ICVT pathophysiology. A major limitation in this case is that we did not screen for all types of inherited thrombophilic factors and test for specific conditions that predispose homocysteinemia.

Given above, we emphasize the need for physicians to be aware of ICVT in the differential diagnoses in patients with risk factors, classical symptoms, and parenchymal brain lesions in or near cortex. In such a case, T2-susceptibility-weighted GE sequence and DSA should be additionally performed to differentiate it from brain tumors. Although the diagnosis is challenging in those without cord sign, meticulous analysis to identify minor but meaningful signs of the thrombosed vein from radiological examinations may aid in reaching a definitive diagnosis of this uncommon entity.

From a pathophysiologic point of view, recanalization of the occluded vein is crucial in preventing the consequences of venous congestion. Thus, treatments for ICVT include the elimination of underlying causes, anticoagulation, and the management of intracranial hypertension. Most neurologists now start with heparin once the diagnosis is determined, even in the presence of hemorrhagic infarct (26), while decompressive craniotomy could be helpful under the condition of progressive neurological impairment or massive parenchymal lesions (19). However, there are several important issues concerning current therapeutic strategies. Firstly, notwithstanding positive results reported, frustrated voices concerning the efficacy of anticoagulation never diminished (9, 19); Secondly, cerebral infarction with hemorrhagic transformation is commonly present at the time of diagnosis of ICVT, which further complicates anticoagulation (9, 19); Thirdly, anticoagulation may not be recommended after craniotomy (irrespective of diagnostic surgery, decompression, or hematoma removal) or brain biopsy in the case of ICVT. Given above, there are several rationales for surgical thrombectomy: (1) to realize definite recanalization and (2) to avoid complications of anticoagulation (e.g., systematic bleeding). Herein, long-term recanalization of the occluded vein was achieved after surgical thrombectomy. Considering these, we recommend surgical thrombectomy in the management of ICVT, especially when craniotomy is performed or a definite diagnosis is made during craniotomy.

In conclusion, this case is unique in two aspects: (1) imaging findings presented unlike those reported in previous cases of ICVT and (2) a good prognosis after surgical thrombectomy. To the best of our knowledge, there is no published report regarding surgical thrombectomy in ICVT. Although observational studies in a larger cohort are urgently needed to confirm the efficacy, we are optimistic that this therapeutic alternative would lead to better outcomes for patients with ICVT.

## Data availability statement

The original contributions presented in the study are included in the article/**Supplementary Material**. Further inquiries can be directed to the corresponding authors.

## Ethics statement

Written informed consent was obtained from the individual(s) for the publication of any potentially identifiable images or data included in this article.

## Author contributions

SF and CY have been involved in the operation and management of the patient. SF and GL designed the report. YB, SF, and GL reviewed the literature and drafted the article. YB and LD prepared the figures. All authors contributed to the article and approved the submitted version. 

## Funding

This research was supported by the National Natural Science Foundation of China (grant No. 81971133 to G.L. and 82101318 to Y.B.) and Science & Technology Plan of Liaoning Province (grant No. 2021JH2/10300116 to S.F.).

## Acknowledgments

We thank Dr XL in the Department of Pathology for her help in neuropathology analysis.

## Conflict of interest

The authors declare that the research was conducted in the absence of any commercial or financial relationships that could be construed as a potential conflict of interest.

## Publisher’s note

All claims expressed in this article are solely those of the authors and do not necessarily represent those of their affiliated organizations, or those of the publisher, the editors and the reviewers. Any product that may be evaluated in this article, or claim that may be made by its manufacturer, is not guaranteed or endorsed by the publisher.
