# Supplementary figures and images for: Case Report: Surgical thrombectomy in a patient with isolated cortical vein thrombosis previously misdiagnosed as brain tumor
Source: Front Oncol. 2022 Nov 1;12:977038. doi: 10.3389/fonc.2022.977038 (PMC9671071; doi:10.3389/fonc.2022.977038)

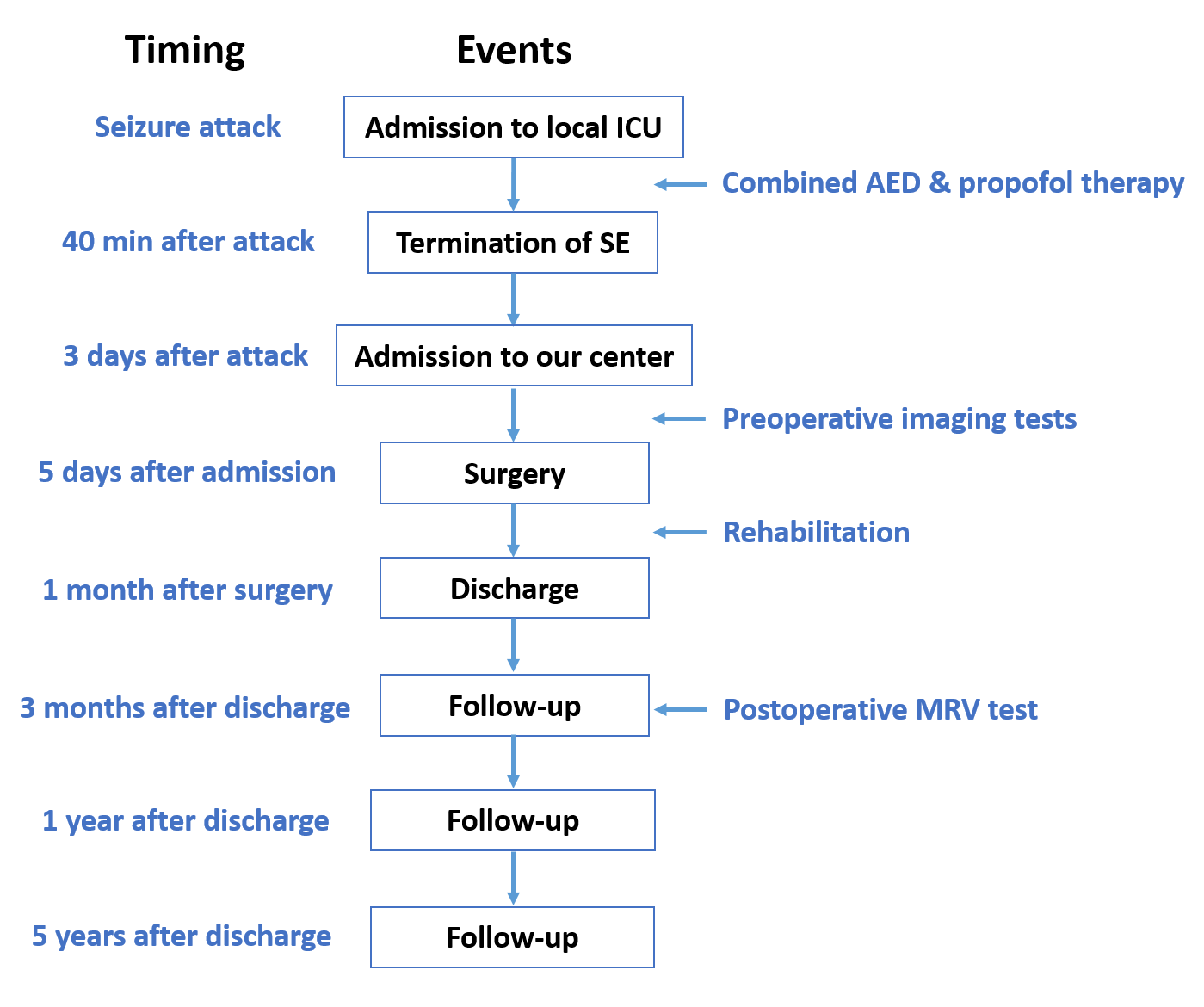

Supplement: Supplementary file 1 [file Image_1.tif]
